# Supplementary material for: Ensuring equitable access to quality HIV care for affected populations in complex sociocultural settings: Lessons from Nigeria
Source: PLoS One. 2025 May 14;20(5):e0319807. doi: 10.1371/journal.pone.0319807 (PMC12077709; doi:10.1371/journal.pone.0319807)
Supplement: Attachment — Submitted filename: Comments_Plos One_2024.docx [file pone.0319807.s002.docx]

This paper provided an account of programme strategies to increase access to HIV prevention (PrEP), testing and treatment services for members of key populations living with HIV (KPLHIV) in northern Nigeria, a context of legal and sociocultural barriers that impede service delivery. This study has the potential to provide evidence and guidance to policymakers, implementers, and practitioners on how to adopt or adapt best practices to meet the needs of KPLHIV.

**General comment**

- This paper is regarded as original research, but the study design, data collection, and analysis are not explained in detail. While the authors mentioned the source of data for the secondary data, the type of data collected (variables) and operational definition of key indicators/outcomes (linkage to care rate) are lacking. Authors should include this information for clarity.
- When introducing acronyms, authors should ensure they are maintained throughout the document for consistency. For example, KPs and KP are used interchangeably to mean Key populations.
- Authors should ensure references (missing citations) are included for data reported and facts.
- Nigeria is a big country with many regions and states. To better understand the context and situate findings, authors should state the specific northern regions (e.g. Northcentral, Northwestern, etc) and states where SFH implemented these novel strategies in Nigeria
- What is the estimated population of KP in northern Nigeria (from KP size estimates, if any) to understand the treatment coverage gap and the extent to which the programme/strategies are closing the gap
- It is not clear whether HIV services were offered to KP in or through the same location or model of care for all KP groups or specific subgroups (FSW or MSM). Are these strategies implemented in the same manner for all KP groups, or are they different per KP group?
- This manuscript is silent on the sub-optimal coverage of viral load testing in the programme. This information is crucial because it reflects on the resources available in the program and access to essential HIV/AIDS services. Contextualize your finding of sub-optimal VL coverage in northern Nigeria despite a seemingly good retention rate (as demonstrated by the increasing number of clients on ART in your programme)
- Expound on the VL testing trend, barriers to accessing VL testing in the programme, and solutions in the discussion section

**Specific comments**

***Abstract (results)***

- Access to viral load testing services is crucial to optimal treatment outcomes. Authors should consider including performance or trend in viral load coverage in their results.

***Main Article***

***Introduction***

- Paragraph 1, lines 2-3 reads: ‘’…..about 39 million people (about twice the population of New York)’’. Since this study is situated in sub-Saharan Africa, perhaps relating this population to a region, country or city in Africa would be more meaningful to readers.
- Paragraph 2, lines 2-4 states: ‘’KPs are defined groups who…..’’ Authors should provide a reference(s) for this definition.
- Provide a reference for the second statement in paragraph two
- Edit decriminalize to criminalize in this statement: ''……for example, Nigeria is among the country that decriminalizes lesbians, gay, bisexual ….(LGBTQI).''

**Methods**

- There is no mention of the specific study design used for this study. Secondary data analysis is not a study design.
- Information about data sources and analysis is coming a bit early. I suggest a subheading titled "data collection and analysis."
- The description of the study area should include the names of states in northern Nigeria where SFH is implementing the KP programme.
  - Refer to KP size estimates in Nigeria (if any) to better understand the context and the extent to which your KP program strategies are closing the treatment gaps.
- While the authors mentioned the source of data for the secondary data, they did not describe the type of data collected (variables) and the operational definition of key indicators/outcomes (linkage to care rate). Authors should include this information for clarity.

***Strategies***

- Authors should consider rephrasing the subheadings for consistency and clarity: rephrase the second strategy to align with the first one. ……’’addressing systems barriers vs addressing structural barriers’’.
- What is retainer-ship? elaborate on this for clarity

***Data management***

- Authors often used adjectives to qualify their project input and activities or processes, such as **robust** data management practices, **high-quality** data, **and highly skilled** data management staff. Although authors operationalize these qualities by describing their unique characteristics or elements, they can be modest in using superfluous adjectives while describing their research.

**Result**

- The authors showed project performance along the cascade of HIV care and trends in HIV case finding, linkage to ART, current on ART, and PrEP uptake. In the method section, authors should describe the data considered and variables collected. Also, definitions for indicators such as HIV positivity rate, linkage to care, and viral suppression rate should be provided.
- The overall viral load coverage is sub-optimal, 69%. Add a comment on this in the results section and discuss the VL coverage trend, barriers to accessing VL testing in the programme, and solutions in the discussion section
- While the number of clients accessing PrEP increased over the study period, authors should consider presenting data on PrEP uptake/coverage among eligible clients (PrEP offer). This is more informative than the number of clients accessing PrEP services in the program.

**Discussion**

- Paragraph 1: reference this statement: ‘’….Presently, several programs focus on drug use…………………harm reduction interventions''.
- For clarity, rephrase this statement: ‘’The …..indicating a potential surge in this sub-population’’. What do authors mean by ‘’surge’’?
- How does the ''surge intervention'' differ from the strategies described in the method section? Expatiate on this
- Many studies/programmes have described sub-optimal viral load coverage in KP programmes but a reasonable viral load suppression rate. This information is essential because it reflects on the resources available in the program and access to critical HIV services. Contextualize your finding of sub-optimal VL coverage in northern Nigeria despite a seemingly good retention rate (as demonstrated by the increasing number of clients on ART in your programme)
  - Discuss the VL trend, barriers to accessing VL testing in the programme, and solutions in the discussion section
- In paragraph 4, the authors write: ‘’…..findings from Portugal buttressed the need for continuous sensitization and education of KPs…..just like we reported in this study’’. Kindly rephrase this statement, as authors did not assess or report the effect of KP sensitization and education on HIV services uptake in this paper/study
- Last paragraph: typographical error----compounders vs confounders

**Figure 1:**

- Introduce legends or labels to show or differentiate strategies from activities
- DSD*= edit to remove or define the asterisk
